# The influence of blood on the efficacy of intraperitoneally applied phospholipids for prevention of adhesions

**DOI:** 10.1186/1471-2482-7-14

**Published:** 2007-07-25

**Authors:** Nick Butz, Stefan A Müller, Karl-Heinz Treutner, Michail Anurov, Svetlana Titkova, Alexander P Oettinger, Volker Schumpelick

**Affiliations:** 1 Department of Surgery, Medical Faculty Rhenish-Westphalian Technical University, Aachen, Germany; 2 Department of Surgery I, Klinikum Mutterhaus der Borromäerinnen, Trier, Germany; 3 Abdominal Center, Park-Klinik Weissensee, Berlin, Germany; 4 Joint Institute for Surgical Research Russian Medical State University, Moscow, Russia

## Abstract

**Background:**

The formation of adhesions following abdominal surgery is a well known problem. In previous studies we demonstrated the efficacy and safety of intraperitoneally applied phospholipids in order to prevent adhesion formation. This study evaluates the influence of blood on the efficacy of intraperitoneally applied phospholipids for prevention of adhesions.

**Methods:**

In 40 Chinchilla rabbits adhesions were induced by median laparotomy, standardized abrasion of the visceral and parietal peritoneum in defined areas of the ventral abdominal wall and the caecum. The animals were randomly divided into four groups. They received either phospholipids 3.0% or normal saline (NaCl 0,9%) (5 ml/kg body weight). In 50% of the rabbits we simulated intraperitoneal bleeding by administration of blood (1,5 ml/kg body weight). The other half served as control group. Ten days following the operation the animals were sacrificed and adhesion formation was assessed by computer aided planimetry and histopathologic examination.

**Results:**

The median adhesion surface area in the NaCl-group (n = 9) amounted to 68,72 mm^2^, in the NaCl+Blood-group (n = 10) 147,68 mm^2^. In the Phospholipid (PhL)-group (n = 9) the median adhesion surface area measured 9,35 mm^2^, in the PhL+Blood-group (n = 9) 11,95 mm^2^. The phospholipid groups had a significantly smaller adhesion surface area (p < 0.05).

**Conclusion:**

Again these results confirm the efficacy of phospholipids in the prevention of adhesions in comparison to NaCl (p = 0,04). We also demonstrated the adhesion preventing effect of phospholipids in the presence of intraperitoneal blood.

## Background

Postoperative peritoneal adhesions are frequent and serious sequelae after abdominal surgery. They cause recurrent and chronic complaints and pain as well as female infertility, and they increase the duration and complication rate of reoperations[1,2]. The incidence of postoperative peritoneal adhesions and their related complications are constantly rising parallel to increasing longevity and growing numbers of surgical procedures[3,4]. Today they are the most frequent cause of intestinal obstruction, account for 3% of all hospital admissions in general surgery, and are responsible for about 30% of all cases of female infertility. Intestinal obstruction as the most life threatening adhesion-related disease is associated with mortality rates of up to 15%[5,6]. Adhesions as almost inevitable sequel after abdominal procedures are generating a rising clinical workload and need for substantial health care expenditures [7-12].

Peritoneal lesions caused by abrasion, ischaemia, desiccation, infection, thermal injury, and foreign bodies are the origin of adhesions. Especially the presence of blood is an initiating causepoint of adhesions. Traumatization leads to a deposition of fibrin and fibrinous, potentially temporary, adhesions. Postoperatively, however, tissue plasminogen activator (t-PA) activity is reduced and inflammatory cytokines (tumor necrosis factor (TNF), Interleukin-1 (IL-1), Interleukin-6 (IL-6)) as well as plasminogen activator inhibitors (PAI-1, PAI-2) are elevated. Due to the hereby decreased fibrinolytic activity in the peritoneal cavity, the fibrin depositions are infiltrated by granulocytes, monocytes, and fibroblasts followed by ingrowth of capillaries, deposition of collagen, and fibrous permanent adhesions[13].

The clinical consequences call for adjuvant means for prevention and control of peritoneal adhesions. A plethora of attempts have been made at prevention and control of postoperative adhesion formation. All efforts directed at the reduction of the surgical trauma by using laparoscopic access and meticulous preparation however, could not solve this problem[14,15]. With the pathogenesis in mind, the idea to enhance the fibrinolytic activity by e.g. urokinase or recombinant tissue plasminogen activator (rtPA) was striking. But in the clinical routine the result would be a possible disturbance of the delicate balance between coagulation and fibrinolysis causing a postoperative bleeding hazard[16].

Non-steroidal anti-inflammatory drugs and corticosteroids carry the risk of haemorrhage, ulcers with subsequent bleeding, immunosuppression, and healing disorders. Saline solutions are too rapidly absorbed and hydrofloatation by macromolecular solutions like dextran show significant adverse effects like fluid shifting, impairment of liver function, even rare cases of dissiminated intravascular coagulation, and anaphylactic shock[15,17].

Phospholipids are a surfactant-like substance with excellent release and lubricating properties that adsorb as an oligolamellar lining to the mesothelium[18]. They offer the possibility to cover the whole surface of the visceral and parietal peritoneum by a small amount of fluid during healing of the serosal defects. By acting as a liquid barrier separating opposite areas of the peritoneum by a very thin membrane-like film they proved to reduce adhesion formation and reformation in different settings including general peritonitis[19,20].

Experiments with different settings demonstrated a significant reduction of adhesion formation after single intraabdominal administration without any adverse effects [19-22].

During operative procedures some degree of bleeding is inevitable. This small amount of blood can induce adhesions. Special barriers for prevention of adhesions in form of patches are ineffective in the presence of blood [29-31]. In this study we evaluate the efficacy of phospholipids in a bleeding model.

## Methods

### Animals and Anaesthesia

A total of 40 Chinchilla rabbits (mean body weight 2.9 ± 0.6 kg) were included in this study. The animals were kept in single cages under standard laboratory conditions with unrestricted access to a balanced pellet diet and water. After adaptation, the rabbits were randomly assigned to four different groups of equal numbers. The surgical procedures were performed under sterile conditions and general anaesthesia by i.v. administration of ketamine (Ketamin^®^10%, Sanofi-Ceva, Düsseldorf, Germany) and xylazine (Xylazin 2%Bernburg, medistar, Germany). The protocol was approved by the local Animal Use and Care committee and the experiments were conducted in accordance with the animal protection laws.

### Induction of Adhesions

Via a midline incision, the caecum was exposed. The parietal and visceral peritoneum of the ventral abdominal wall and the caecum, respectively, were abraded within a total area of 9 cm^2^. For this purpose a stamp calibrated to a pressure of 4 kPa with an emery paper (260 grains per cm^2^) mounted to a curved plate was used[22].

### Experimental Groups

The animals were randomly assigned to one of four groups. We either applied phospholipids 3.0% or normal saline (NaCl 0,9%) (5 ml/kg body weight) to the peritoneal cavity. In half of the rabbits of each group we simulated intraperitoneal bleeding (1,5 ml blood/kg body weight) as described below. The other half received no additional treatment. (see table 1 + 2)

**Table 1:** animals/group

	**∅**	**Blood**
**Control (NaCl)**	9	10
**Phospholipids (PhL)**	9	9

**Table 2:** treatment/group

***Blood***	***NaCl***	***PhL***	***Group name***
	X		NaCl
		X	PhL
X	X		NaCl + Blood
X		X	PhL + Blood

In the bleeding groups the blood was taken from a mesenterial vene in a dosage of 1.5 ml per kg body weight. The blood was dispersed in the abdominal cavity near to the abraded areas (described above). Then the midline incision was closed by a running suture 3-0 polyglycolic acid.

Prior to the last stitch of abdominal closure 5 ml per kg body weight of phospholipids (Fresenius, Germany) or 0.9% saline solution (NaCl, control group) were instilled into the abdominal cavity. The lipid emulsion consisted of purified egg derived phospholipids, stabilized by glycerol, adjusted to physiological pH by sodium hydroxide, and water for injection to achieve a concentration of 3.0% (30 g phospholipids per 1000 g of emulsion). The final product was supplied in sterile and pyrogene-free condition. Evaluation of all parameters was carried out in a blinded fashion.

### Assessment of Adhesions

After 10 days (n = 37) the animals were sacrificed by a lethal dose of pentobarbital sodium (Narcoren^®^, Rhône Merieux, Laupheim, Germany). The abdomen was opened via a full left para-median incision for complete exploration. Peritoneal adhesions were meticulously dissected and the intestine was opened along the mesenteric attachment for computed planimetry.

### Histology

Adhesion carrying tissues (bowel, abdominal wall, omentum) were excised en-bloc and fixed in formaldehyde solution. Sections with a thickness of 5 μm were stained with haematoxylin and eosin (HE) for light microscopy to evaluate the structure of the connective tissue and the healing process.

### Statistical Analysis

All data are expressed as median. The Mann-Whitney U test for independent samples was used for statistical analysis. P < 0.05 was considered statistically significant.

## Results

Performance of the initial surgical procedure was uneventful in all animals. Postoperatively three animals of three different groups died of pneumonia. Therefore the following data are based on the final examination of 37 animals.

The median (in mm^2^) of the adhesion area in the NaCl group (n = 9) was 68,72 and in the NaCl+blood group 147,68.

**Figure 1:**
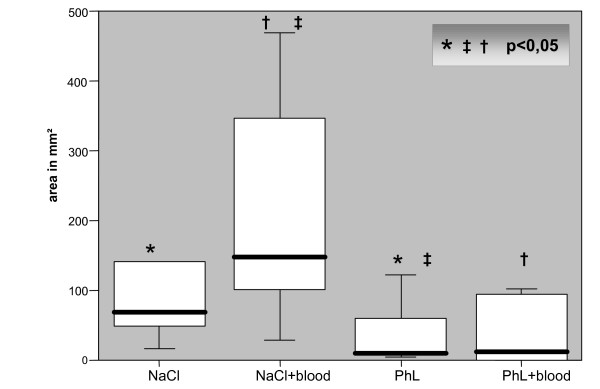
median (range) of adhesion area/group (Box-and Whisker-Plot).

In contrast to these results the median of adhesion area in the PhL group was only 9,35 mm^2 ^and in the PhL+blood group 11,95. (see Figure 1)

The adhesion area in the NaCl group is significantly higher compared to the PhL group (p = 0,04). Between the blood groups again the one with added NaCl showed a significantly higher adhesion area compared to the one with added PhL (p = 0,017). Consequently the adhesion area in the NaCl+blood group compared to the PhL group is also significantly higher (p = 0,004).

No statistically significant difference was seen between the two PhL groups (p = 0,863). (see Figure 1)

Histological analysis of representative specimens of the injured sites revealed an inflammatory-reparative tissue response with non-specific granulation tissue infiltrated by mono- and polymorphonuclear cells. Activated mesothelial cells were found on the serosal surface as well as subserosal fibroblastic proliferations. No differences of the histological and cellular patterns were observed between the control and the test groups.

## Discussion

During visceral surgery damage to the mesothelial lining is inevitable due to desiccation, thermal injury and surgical trauma. These lesions lead to oozing out of a fibrinous exsudate, a reduction of fibrinolytic acivity and subsequent adhesion formation [23-25].

The refinement of surgical technique, minimizing the peritoneal injury and avoidance of tissue ischaemia markedly reduce postsurgical adhesions, but do not dissolve them completely.

Previous studies have shown the beneficial effect of phospholipids in preventing adhesion formation in a standard visceral surgery setting in rabbits[21,22,26].

Phospholipids, polar phosphoric acid di-esters are the natural constituents of abdominal cavity fluid and cell membranes. Human mesothelial cells were found to rapidly synthesize and secrete this surfactant-like substance. Phospholipids are zwitterions with a positive charged quaternary ammonium ion that is able to bind to negative charges of epithelial surfaces [27]. This fact enables small amounts of phospholipids to cover the whole surface of the visceral and parietal peritoneum during the healing process of serosal defects in the surgical setting[28].

By mechanical separation of the two healing surfaces phospholipids are able to reduce adhesion formation in different settings[20,21].

In our study we investigated the influence of peritoneal bleeding on adhesion formation in the presence of phospholipids. Few data exist with regard to the latter.

Bioresorbable membranes like Seprafilm^® ^or Interceed^® ^showed in one study a reduced effectiveness in the prevention of adhesions in the presence of blood in the abdominal cavity.[29,30] In the case of Interceed barrier (oxidized regenerated cellulose) the presence of blood abolished its effect on the prevention of coecal adhesions and all in all the effectiveness was reduced except in neutralized Interceed (nTC7)[31].

For the hyaluronan derivative gel (auto-cross-linked polysaccharide (ACP) gel) in different preparations (20,40,60 mg/ml) De Iaco et al. demonstrated the effectiveness in postsurgical adhesion prevention even in the presence of blood. There were no differences between the phospholipid groups[32].

Our results are in line with our previous studies and confirm the efficacy of phospholipids in adhesion prevention compared to a control group (p = 0,04).

The mean adhesion area counts to 9,35 mm^2 ^in PhL and 11,95 mm^2 ^in the PhL+blood group. In addition the effectiveness of PhL in adhesion prevention was not reduced by the presence of intraabdominal blood.

## Conclusion

Again, these findings demonstrate the significant capability of phospholipids to reduce adhesion formation. The presence of intraabdominal blood does not interfere with its efficacy.

## Competing interests

The author(s) declare that they have no competing interests.

## Authors' contributions

NB has made substantial contributions to conception and design, acquisition of data, analysis and interpretation.

SM has made substantial contributions to conception and design, has been involved in drafting the manuscript and performed the statistical analysis.

KHT has been involved in drafting the conception and revised the manuscript critically.

MA and ST have made substantial contributions to acquisition of data and analysis.

APÖ and VS have made substantial contributions to conception and design and read and approved the final manuscript.

## Pre-publication history

The pre-publication history for this paper can be accessed here:


